# Hepatic inflammatory pseudotumor-like follicular dendritic cell tumor: a case report

**DOI:** 10.1186/s13256-021-02957-5

**Published:** 2021-07-29

**Authors:** Ana Daniela Pascariu, Andreea Ioana Neagu, Andrei Valentin Neagu, Alexandru Băjenaru, Cezar Iulian Bețianu

**Affiliations:** 1 Department of Radiology, “Carol Davila” Central Military Emergency University Hospital, Mircea Vulcanescu Street, no. 88, 010825 Bucharest, Romania; 2 Department of Interventional Radiology, “Carol Davila” Central Military Emergency University Hospital, Mircea Vulcanescu Street, no.88, 010825 Bucharest, Romania

**Keywords:** Epstein–Barr virus, Follicular dendritic cell sarcoma, Hepatic tumor, Inflammatory pseudotumor, Case report

## Abstract

**Background:**

Inflammatory pseudotumor-like follicular dendritic cell sarcoma is a rare histological variant of follicular dendritic cell sarcoma involving typically the spleen and the liver, often linked to the presence of Epstein–Barr virus infection. Definite diagnosis of this type of sarcoma is difficult to make owing to nonspecific clinical and imaging findings and is based on histopathological features. Inflammatory pseudotumor-like follicular dendritic cell sarcoma is described as a low-aggressivity tumor with a favorable prognosis.

**Case presentation:**

We report the case of a 34-year-old Caucasian woman, Epstein–Barr virus positive, diagnosed with hepatic inflammatory pseudotumor-like follicular dendritic cell sarcoma and surgically treated in November 2014, who developed 6 years later a recurrence for which she underwent once again surgical treatment. As far as we know, fewer than 30 reports of inflammatory pseudotumor-like follicular dendritic cell liver tumors have been reported in the English literature.

**Conclusions:**

Although it is an uncommon tumor, inflammatory pseudotumor-like sarcoma is a diagnostic worth being taken in consideration, and surveillance is recommended owing to the possibility of recurrence.

## Introduction

Follicular dendritic cell (FDC) sarcoma is a rare, low-grade neoplasm originating from follicular dendritic cells, which are antigen-presenting cells located in the germinal centers of lymphoid follicles [1]. FDC sarcomas mainly occur in the lymph nodes, with only one-third of cases affecting extranodal sites such as the gastrointestinal tract, soft tissue, or skin [2]. FDC sarcoma of the liver represent < 0.1% of all primary hepatic tumors [3]. Histologically, FDC sarcomas can be classified into two types: conventional FDC sarcoma and inflammatory pseudotumor-like follicular dendritic cell (IPT-like FDC) sarcoma. IPT-like FDC sarcoma presents morphological and clinical characteristics intermediate between inflammatory pseudotumor and FDC tumor and was first described in 2001 by Cheuk *et al*. [4]. IPT-like FDC sarcoma is a very uncommon entity, with a favorable prognosis, located typically in the spleen and the liver, usually associated with Epstein–Barr virus (EBV) [4]. Due to scarcity of the cases and lack of specific clinical and imaging features, the diagnosis of IPT-like FDC sarcoma is problematic. Therefore, we report the case of a young woman, diagnosed with hepatic IPT-like FDC sarcoma, who developed a recurrence after the initial surgical treatment.

## Case presentation

A 34-year-old Caucasian woman, complaining of epigastric pain, was referred to our department in October 2014 for further evaluation and treatment, after a liver lesion was found on an ultrasound examination. She had no prior significant disease history.

The laboratory data values obtained were all normal, except for an elevated serum level of fibrinogen 552 mg/dl (normal, 150–400 mg/dl). Serological tumor markers such as alpha-fetoprotein, carcinoembryonic antigen, CA125, and CA19-9 were not measured.

**Fig. 1:**
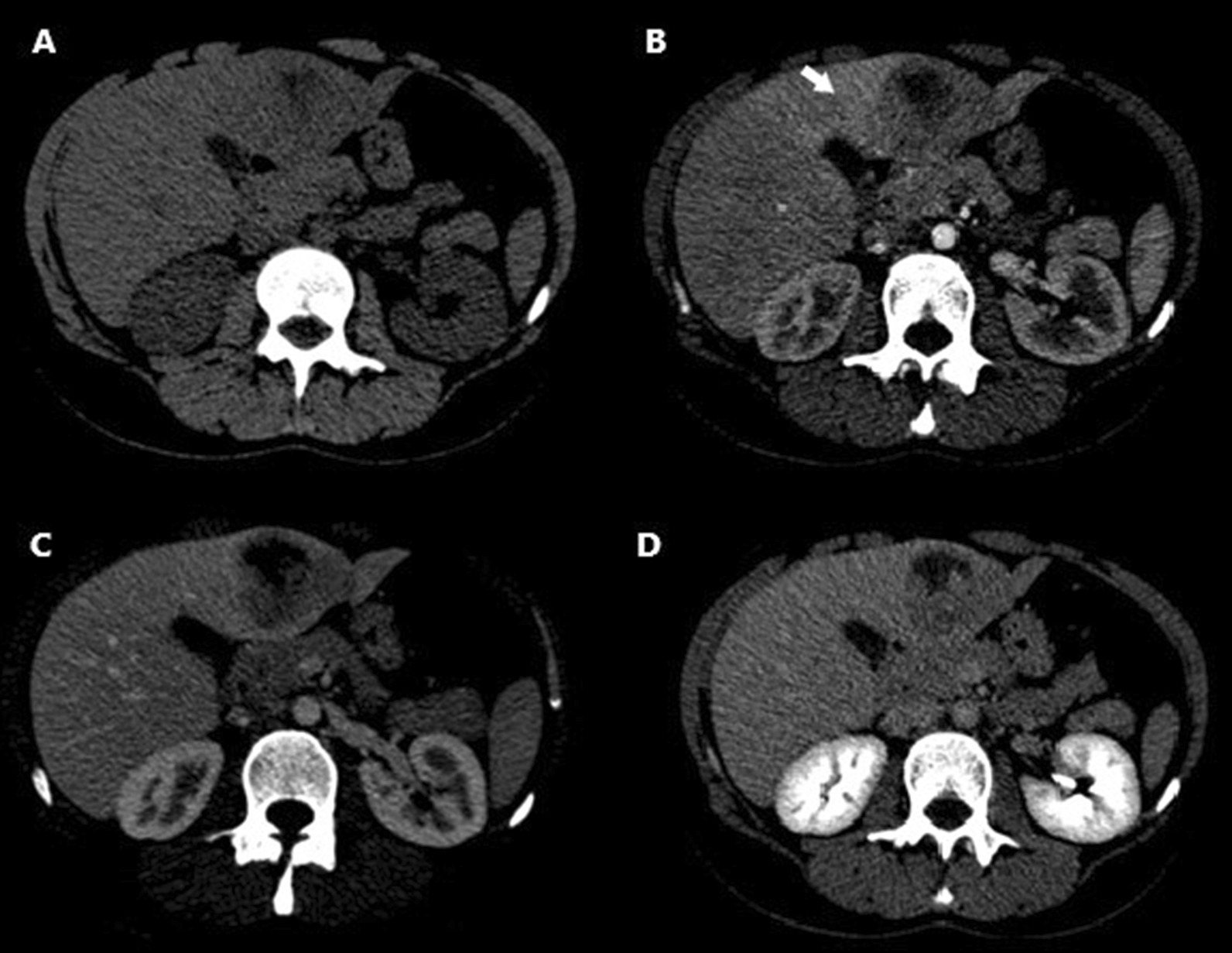
**A** Nonenhanced CT showing a heterogeneous hypodense mass, located in segment IVB of the liver. **B**–**D** Multiphase enhanced CT: arterial (**B**), venous (**C**), and delayed phases (**D**) revealing heterogeneous sustained hypoenhancement of the hepatic tumor. **B** Arterial phase demonstrating left lobe perfusion disorder (white arrow)

In terms of imaging examinations, only a contrast-enhanced computed tomography (CT) was performed, unfortunately. Nonenhanced abdominal CT showed a heterogeneous hypodense mass, located in segment IVB of the liver. Contrast-enhanced CT revealed one slightly well-defined tumor, measuring 60/55 mm in diameter with heterogeneous sustained enhancement due to central necrosis (Fig. 1). Another CT finding was left liver lobe periportal infiltration with associated transient enhancement during the arterial phase (hepatic perfusion disorder). No evidence of lymph node enlargement was present in the abdomen or pelvis. Correlating the clinical features (age of the patient, oral contraceptive use, lack of systemic symptoms, normal laboratory results) with the radiological findings, the diagnosis was orientated towards a benign hepatic tumor (hepatic adenoma or inflammatory pseudotumor).

The patient underwent successful laparoscopic removal of the tumors. The resected specimens were submitted for histopathological examination and revealed a well-demarcated infiltration of a large number of lymphocytes, plasma cells, and spindle cells (Fig. 3A) . The spindle cells showed no pleomorphism and were scattered; some of them were arranged in disordered bundles with whorled, storiform, and sheet pattern, with unclear boundaries with pale or eosinophilic cytoplasm and larger nuclei than normal (Fig. 3B). Immunohistochemical staining showed strong positive expression of Clusterin and EBV- LMP1 and diffuse positivity for CD21(+) and CD35(+), and Ki-67 index of (5–7%+). The positive expression of CD5, CD7, and CD138 highlighted lymphocytes and plasma cells and also CD20 positive in B cell in the background. S-100(−) and CD34(−) were negative. The histopathological diagnosis was EBV-associated IPT-like-FDC sarcoma of the liver.

The postoperative course was uneventful, and it was decided that the patient would be monitored annually by magnetic resonance imaging (MRI). Six years later, after the surgery, the patient had a recurrence that was detected on contrast-enhanced magnetic resonance imaging (MRI).

**Fig. 2:**
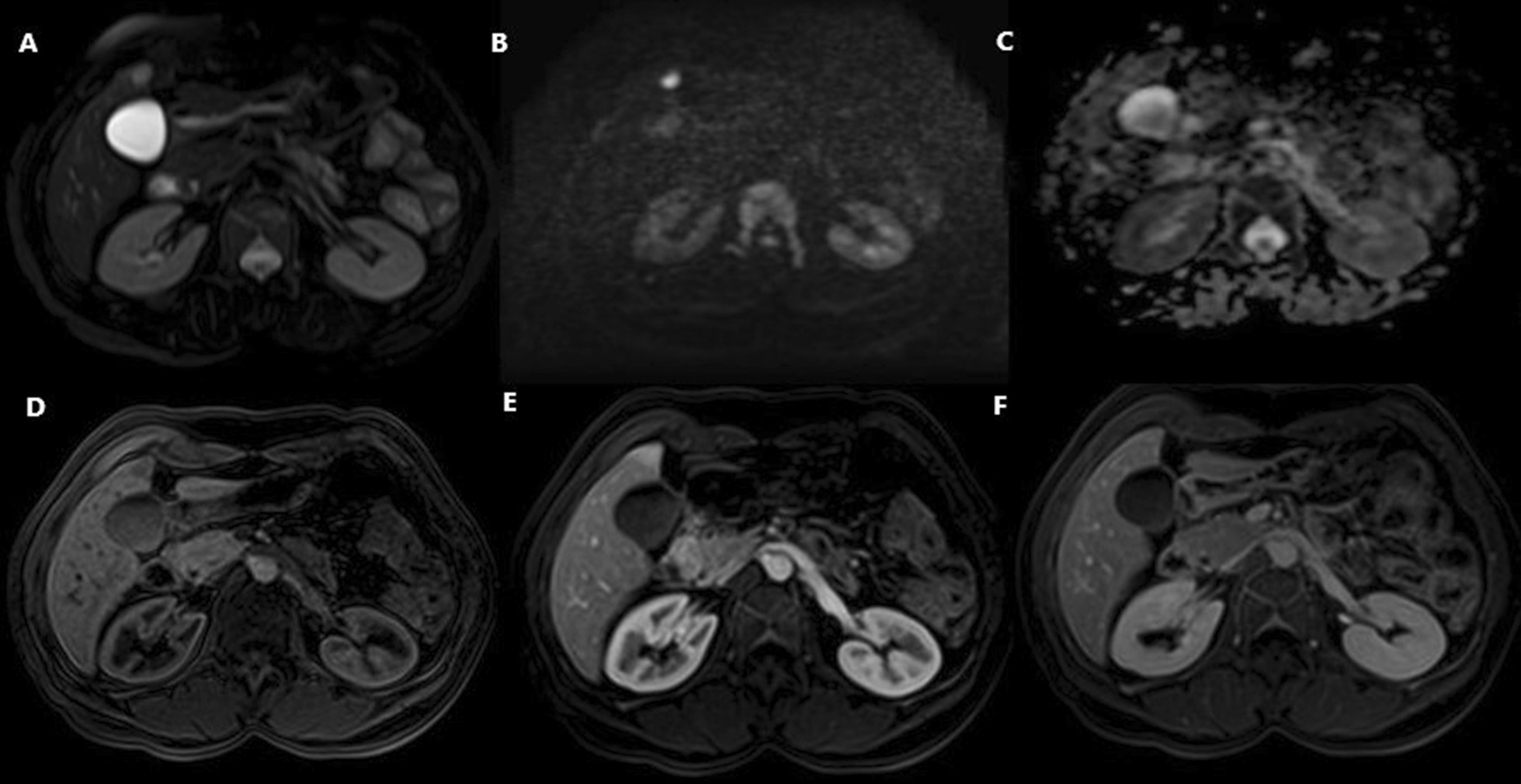
**A**–**C** MRI showing a well-defined lesion in IVB segment hyperintense T2 fat suppressed (**A**) demonstrating restriction to diffusion (**B**, **C**) with delayed enhancement on contrast-enhanced images (**D**–**F**).

The abdominal MRI revealed a well-defined, nodular lesion in IVB segment, measuring 12 mm in longest diameter, hypointense T1, hyperintense T2, hyperintense on diffusion-weighted imaging (DWI), and hypointense on apparent diffusion coefficient (ADC) showing restriction to diffusion (Fig. 2). The blood tests were within normal limits, and the patient was asymptomatic. Once again, surgical excision of the lesion trough laparoscopic approach was opted out. The postoperative histopathological diagnosis and immunohistochemical staining strongly support the recurrence of the hepatic EBV-associated IPT-like-FDC sarcoma. The patient recovered well, and no metastases or recurrence had developed during the 2-month follow-up.

## Discussion

Inflammatory pseudotumor-like FDC sarcoma is a histological variant of FDC sarcoma, which consists of a large number of neoplastic spindled cells with a certain degree of atypia that exist in a inflammatory background which contains lymphocytes and plasma cells [4]. The immunophenotype of IPT-like FDC sarcoma is the same as that of FDC sarcoma: positivity for Clusterin CD21, CD23, and CD35, and in addition positivity for EBV [5].

IPT-like sarcoma mostly affects the female population (2–3:1), and the median age for diagnosis is 57 years [6]. IPT-like FDC sarcoma has an extremely low incidence and usually involves the spleen and the liver, but colonic and pancreatic involvement are also described [7, 8]. As far as we know, there have been fewer than 30 reports of IPT-like FDC liver tumors in the English literature [9, 10].

Regarding the clinical presentation of IPT-like FDC sarcoma, it may vary from asymptomatic patients to abdominal discomfort and pain, abdominal distension, fever, and weight loss [6].

The radiologic findings of IPT-like FDC tumors were summarized by Hai-Lan *et al*. and showed heterogeneous soft-tissue density with sustained hypoenhancement on CT examination [10]. Central necrosis or hemorrhage may be present [11].

**Fig. 3:**
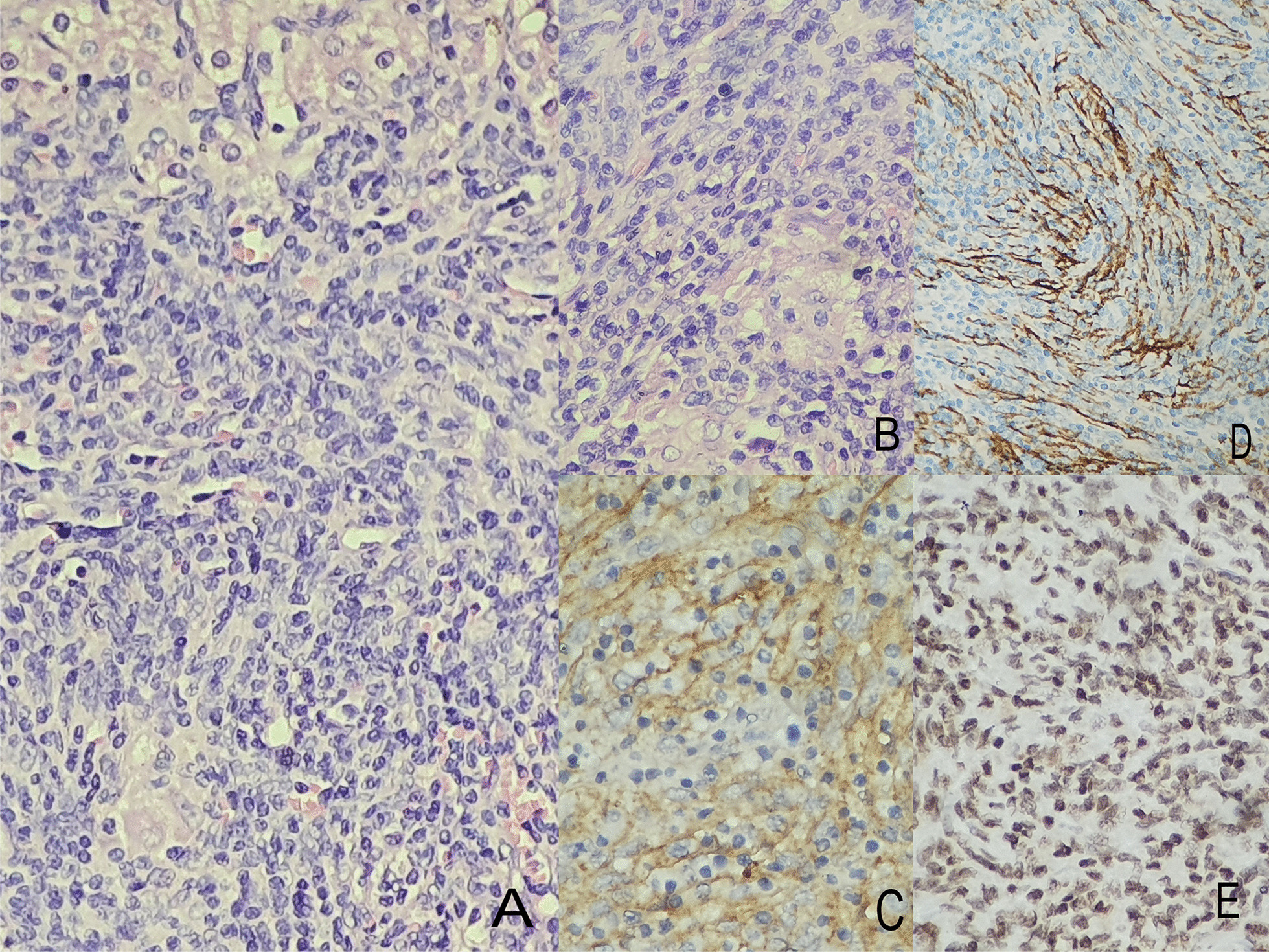
**A** Hematoxylin–eosin stain ×20 liver parenchyma (top), with intercalated bundles and a dense inflammatory infiltrate that includes lymphocytes (bottom). **B** Hematoxylin–eosin stain ×40 showing tumor mass consisting of a dense inflammatory infiltrates, with storiform pattern composed of oval cells with enlarged, vesicular nuclei and distinct nucleoli. **C** Neoplastic cells presenting diffuse positivity for Clusterin (×20). **D** CD 21 is expressed in follicular dendritic cells (20×). **E** Intense positivity for EBV-LMP1 (×40)

Inflammatory pseudotumor-like follicular dendritic cell tumor was proven to be a distinct entity even if it was included in the group of inflammatory pseudotumors. It is localized exclusively in the liver and spleen, and it is related to Epstein–Barr virus (EBV). The neoplastic cells are positive for Clusterin (Fig 3C), follicular dendritic cells (FDC) markers such as CD21 (Fig. 3D), CD23, and CD35, and EBV- LMP1 (Fig. 3E). These play an important role in the diagnosis and differential diagnosis. This panel should be used in correlation with clinical, laboratory, and topographic features to provide a correct diagnosis.

Differential diagnostic of IPT-like FDC sarcoma should take into consideration the following entities: FDC sarcoma, inflammatory pseudotumor (IPT), inflammatory myofibroblastic tumor (IMT), diffuse large B-cell lymphoma, or anaplastic large cell lymphoma.

In hepatic IPT-like FDC sarcoma, EBV infection occurs before the monoclonal proliferation of neoplastic cells. Most spindle cells are EBV positive, but the tumor cells in classic FDC sarcoma are usually EBV negative.

The morphology of inflammatory pseudotumor (IPT) is similar to that of IPT-like FDCS, consisting of spindle cells with almost the same immunohistochemical panel, but not EBV. EBEV ISH and EBV-LMP1 markers are key for differential diagnosis. [12]

Clinical background correlates with immunohistochemical findings to outline the final diagnosis. So far, no standard treatment has been established. Surgical resection is the treatment of choice for patients with localized hepatic lesions [13]. Given the possibility of recurrence, surveillance is recommended [14]. The role of adjuvant therapy has not been well defined.

## Conclusion

Inflammatory pseudotumor like follicular dendritic cell sarcoma is an exceptionally rare tumor with favorable prognosis and nonspecific clinical and imaging manifestations. Definite diagnosis relies on histopathology and immunohistochemistry staining. To avoid the misdiagnosis of liver tumors, IPT-like sarcoma is a diagnostic worth being taken into consideration.

Written informed consent was obtained from the patient for publication of this case report and any accompanying images. A copy of the written consent is available for review by the Editor-in-Chief of this journal.

## Data Availability

Data sharing not applicable to this article as no datasets were generated or analyzed during the current study.

## Abbreviations

FDC sarcoma: Follicular dendritic cell sarcoma
IPT-like FDC sarcoma: Inflammatory pseudotumor-like follicular dendritic cell sarcoma
EBV: Epstein–Barr virus
CT: Computed tomography
MRI: Magnetic resonance imaging
DWI: Diffusion-weighted imaging
ADC: Apparent diffusion coefficient
IPT: Inflammatory pseudotumor
IMT: Inflammatory myofibroblastic tumor
